# A Sanguinarine Analogue Targeting ROS Signaling Exhibits Anti-Tumour Effects by Inducing Apoptosis and Ferroptosis in Osteosarcoma

**DOI:** 10.3390/antiox15080941

**Published:** 2026-07-29

**Authors:** Hui Zhang, Fangjun Cao, Jiaxin Wang, Chenxi Yun, Airong Qian, Xiao Lin

**Affiliations:** 1 Key Lab for Space Biosciences and Biotechnology, School of Life Science and Technology, Northwestern Polytechnical University, Xi’an 710072, China; 2020264680@mail.nwpu.edu.cn (H.Z.); wangjiax@mail.nwpu.edu.cn (J.W.); chenxiyun@mail.nwpu.edu.cn (C.Y.); 2 Advanced Power Research Institute of Northwestern Polytechnical University in Sichuan Tianfu New Area, Chengdu 610213, China; 3 Lab for Bone Metabolism, Xi’an Key Laboratory of Special Medicine and Health Engineering, School of Life Sciences and Technology, Northwestern Polytechnical University, Xi’an 710072, China; 4 Shaanxi Institute of Zoology, Xi’an 710032, China; fang198789@xab.ac.cn

**Keywords:** osteosarcoma, sanguinarine analogue, apoptosis, ferroptosis, ROS, NRF2/GPX4

## Abstract

Osteosarcoma is a common malignant bone tissue tumour that frequently occurs in the adolescent population, with recurrence and metastasis rates that remain high under current treatment strategies, necessitating the development of cost-effective and efficient anti-osteosarcoma drugs. Sanguinarine, a benzophenanthridine alkaloid derived from plants in the Papaveraceae family, possesses anti-tumour capabilities; however, its clinical application is limited due to poor water solubility and potential organ toxicity. Through screening 30 sanguinarine analogues, in this study we found that analogue 25 exhibited significant activity and could suppress cell proliferation and arrest the cell cycle at the G0/G1 phase in U2OS and MG63 cells, with IC_50_ values of 1.484 and 1.954 μM, respectively. Analogue 25 could also induce cell apoptosis by increasing the levels of cleaved caspase-9 and BAX. Subsequently, we demonstrated that analogue 25 increased the ROS level, decreased the mitochondrial membrane potential, and increased lipid peroxidation in osteosarcoma cells. After reducing ROS levels through NAC, analogue 25 was able to regulate mitochondrial-mediated osteosarcoma cell apoptosis by modulating the ROS level. Furthermore, analogue 25 could also regulate the expression of ferroptosis-related proteins (including GPX4, SLC7A11 and PTGS2) through the NRF2/GPX4 pathway, ultimately leading to the accumulation of lipid peroxidation and ferroptosis. In a xenograft mouse tumour model derived from osteosarcoma cell lines, analogue 25 exhibited stronger anti-tumour activity than sanguinarine by regulating apoptosis and ferroptosis without causing liver or kidney toxicity. These findings highlight that sanguinarine analogue 25 has beneficial effects in treating osteosarcoma.

## 1. Introduction

Osteosarcoma is a prevalent malignant tumour observed in children, adolescents, and young adults, constituting approximately 56% of all bone tissue tumours [1] and having a high rate of disability and mortality. The current treatment strategies include surgical resection and neoadjuvant chemotherapy, which can increase the 5-year overall survival rate for osteosarcoma patients to 60% to 70% [2]. When patients develop resistance to chemotherapy or radiotherapy, the osteosarcoma recurrence or metastasis rate can exceed 30%, and the 5-year overall survival rate for these patients has dropped to 20–30% [3]. Given the current challenges faced by traditional treatment methods, developing low-dose yet highly effective drugs is of vital importance for improving treatment outcomes in patients with osteosarcoma.

Natural products provide inspiration for developing new drugs to treat tumours [4]. Due to their diverse chemical structures and ability to undergo various regulatory processes, they have unique advantages in terms of low toxicity and therapeutic efficacy [5]. The natural chemical compounds extracted from plants have been extensively studied in the field of tumour treatment, finding that they can regulate the proliferation, invasion, metastasis, and angiogenesis of tumour cells during tumour occurrence and development, and exhibit excellent anticancer activity [6,7,8]. Sanguinarine is a natural product belonging to the benzophenanthridine alkaloid family which can be extracted from various plants of the Papaveraceae family, such as the *Sanguinaria canadensis* and *Argemone mexicana* plants [9,10]. Many studies have shown that sanguinarine has anticancer effects on hepatocellular [11] and oral squamous cell carcinoma [9], along with breast [12], prostate [13], gastric [14], lung [10], and other cancers. To improve water solubility, stability, and biological activity, a range of sanguinarine derivatives have been designed and synthesized. Previous findings suggest that sanguinarine derivatives 26 (2-(2,4-Dichlorophenyl)phthalazin-2-ium Bromide) and 27 (2-(2,5-Dichlorophenyl)phthalazin-2-ium Bromide) exhibit high biological activity in fungi, inhibiting the structure of fungal hyphae and cell membranes [15]. However, the anticancer effects and mechanisms of sanguinarine derivatives remain unclear.

Excessive or insufficient levels of reactive oxygen species (ROS) are closely related to the pathogenesis of various diseases such as cancer, neurodegenerative diseases, rheumatoid arthritis, and diabetes [16,17]. Cells maintain dynamic redox state balance through the endogenous antioxidant system: when the redox state changes and antioxidants are depleted, oxidative stress will be triggered and oxidative damage will occur [18]. Apoptosis is a form of programmed cell death driven by energy, wherein ROS may cause lipid peroxidation of the inner mitochondrial membrane, thereby triggering apoptotic signal generation. Cytochrome c is further released from the mitochondria into the cytoplasm, leading to caspase-3 activation and apoptosis initiation [18]. The intracellular metabolic pathways, such as respiration and lipid metabolism, can generate a large number of ROS, which can lead to ferroptosis [19], a form of iron-dependent cell death, characterized by lipid peroxidation accumulation and decreased glutathione peroxidase 4 (GPX4) activity. The classical regulatory pathway of ferroptosis is the system Xc/glutathione/GPX4 axis [20], wherein system Xc consists of two subunits, SLC7A11 and SLC3A2, and can transport cysteine into the cell to participate in glutathione (GSH) synthesis. Glutathione peroxidase (GPX) further participates in this process and reduces ROS. The inhibition of system Xc activity can lead to a decline in cellular antioxidant capacity and lipid peroxidation, and ultimately the occurrence of ferroptosis through this signalling pathway [21]. It was reported that Nuclear factor erythroid 2-related factor 2 (NRF2) is vital for the anti-ferroptosis response, as it regulates enzymes like SLC7A11 and GPX4 that are key to GSH synthesis and metabolism [22,23]; therefore, targeting ferroptosis through NRF2/GPX4 presents a new strategy to destroy cancer cells.

The 30 sanguinarine analogues previously synthesized were strategically designed based on the natural sanguinarine and retained the C=N+ bond as the decisive functional group for biological activity, and featured the advantages of simple synthesis and good chemical stability [15]. However, the impact of these sanguinarine analogues on osteosarcoma cells has yet to be elucidated. Consequently, this study aims to identify active compounds that induce apoptosis in osteosarcoma cells. Subsequent investigations will be performed using U2OS and MG63 cell lines to assess their impact on cell proliferation, cell cycle progression, and underlying mechanisms of cell death through both in vivo and in vitro experiments. The findings of this research are expected to offer a theoretical foundation and novel insights for the development of therapeutic agents for osteosarcoma treatment.

## 2. Materials and Methods

### 2.1. Reagents

McCoy’s 5A and Minimal Essential Medium (MEM) were purchased from Procell Life Science &Technology Co., Ltd. (Wuhan, China); fetal bovine serum (FBS) from BI (Biological Industries, Beit Haemek, Israel); dimethylsulfoxide (DMSO) from Sigma-Aldrich (St Louis, MO, USA); antibodies against caspase-9 (sc-133109), Bcl-2 (sc-7382), GPX-4 (sc-166570), PTGS2 (sc-19999), and GAPDH (sc-166574) from Santa Cruz Biotechnology (Dallas, TX, USA); 3-(4,5-dimethylthiazol-2-yl)-2,5-diphenyl-tetrazolium bromide (MTT), Hoechst, Annexin V-FITC Apoptosis Assay Kit, Cell Cycle and Apoptosis Analysis Kit, Reactive Oxygen Species Assay Kit, mitochondrial membrane potential assay kit, Lipid Peroxidation Assay Kit, GSH and GSSG Assay Kit, DAB Horseradish Peroxidase Colour Development Kit, paraformaldehyde, Hematoxylin and Eosin (H&E) Staining Kit, horseradish peroxidase (HRP)-conjugated IgG, RIPA Lysis Buffer and Triton X-100 from Beyotime Biotechnology (Shanghai, China); and FerroOrange solution from MCE (Shanghai, China).

### 2.2. Cell Culture

The osteosarcoma cell lines U2OS and MG63 (Newgainbio, Wuxi, China) were cultured in McCoy’s 5A and MEM supplemented with 10% fetal bovine serum (FBS, Gibco, Waltham, MA, USA), 100 U/mL penicillin and 100 μg/mL streptomycin, respectively. The cells were incubated at 37 °C in a 5% CO_2_ incubator.

### 2.3. Cell Viability Assay

U2OS and MG63 cells (3 × 10^4^ cells/well) were cultured in 96-well plates with 100 μL complete medium for 24 h; then, sanguinarine analogues at different concentrations were added for 48 h. A total of 20 μL MTT solution (5 mg/mL) was added to each well; then, the wells were incubated for 4 h at 37 °C in the dark. The supernatant was removed and 120 μL DMSO was added to each well to fully dissolve the crystals. After incubation at 37 °C for 10 min with shaking, the absorbance value at 570 nm was detected by a Bio-Red microplate reader (Hercules, CA, USA).

### 2.4. Colony Formation Assay

U2OS and MG63 cells (5 × 10^2^ cells/well) were seeded into 6-well plates for 24 h, and then the culture medium was discarded and complete medium containing analogue 25 (2 μM and 4 μM) was added. The analogue was changed every 3 days, and after 7 days of culture, visible cell clones were observed. After washing with PBS 3 times, methanol was added to the cells for 15 min. Then, the fixing solution was removed, and 1% crystal violet staining solution was added to perform staining for 30 min. The cells were finally washed 3 times with PBS and photographed under an inverted microscope (Novel, Ningbo, China).

### 2.5. Annexin V-FITC/PI Assay

The cell apoptosis rate was detected using the Annexin V-FITC Apoptosis Assay Kit (Beyotime Biotechnology, Shanghai, China) according to the manufacturer’s instructions. The osteosarcoma cells were cultured with analogue 25 (2 μM and 4 μM) for 48 h, and 1 × 10^5^ cells were collected and resuspended with 195 μL Annexin V-FITC binding solution. Then they were gently mixed with 5 μL Annexin V-FITC and 10 μL PI staining solution. The cells were incubated for 15 min in dark, and then immediately detected by flow cytometry. The results were analyzed using FlowJo V10 software.

### 2.6. Western Blotting

Proteins were extracted from cells using RIPA lysis buffer and then blotted on polyvinylidene fluoride membranes to prepare whole-cell lysates for Western blotting. The membranes were incubated with antibodies against Pro-caspase9 (1:1000), cleaved caspase9 (1:1000), Caspase3 (1:1000), BCL-2 (1:1000), PTGS2 (1:1000), SLC7A11 (1:1000), GPX4 (1:1000), and GAPDH (1:5000), and secondary antibodies conjugated with HRP (1:2000) were used for signal detection. The blots were visualized using ECL chemiluminescence reagents (Tanon, Shanghai, China).

### 2.7. Cell Cycle Detection

The cell cycle was detected using the Cell Cycle and Apoptosis Analysis Kit (Beyotime Biotechnology, Shanghai, China) according to the manufacturer’s instructions. The osteosarcoma cells were treated with analogue 25 (2 μM and 4 μM) for 48 h, and then collected and resuspended in precooled 70% ethanol at 4 °C for 24 h. After centrifuging at 1000× *g* for 5 min, PI staining solution was added to the cell precipitate, which was then suspended and bathed at 37 °C for 30 min in the dark. The cell cycle was detected using flow cytometry (BD FACSCalibur, Franklin Lakes, NJ, USA), and the cell DNA content was analyzed by FlowJo V10 software.

### 2.8. Quantitative Real-Time PCR (qRT-PCR)

Total RNA was extracted from cells using E.Z.N.A.^®^ Total RNA Kit II kit (Omega Bio-tek, Norcross, GA, USA), and cDNA was obtained by reverse transcription using 5 × Evo M-MLV RT Master Mix (Accurate Biotechnology, Changsha, China). Specific genes for qRT-PCR were detected by 2 × SYBR Green qPCR Master Mix, with the relative amount of gene expression normalized to GAPDH. The primer sequences used for qRT-PCR are listed in Table 1, and all primers were purchased from Tsingke Biotechnology (Beijing, China).

### 2.9. Reactive Oxygen Species (ROS) Assay

The intracellular ROS were detected using the Reactive Oxygen Species Assay Kit with Diluent (Beyotime Biotechnology, Shanghai, China) according to the manufacturer’s instructions. The osteosarcoma cells were cultured with analogue 25 (2 μM and 4 μM) for 48 h, and after discarding the culture medium, 10 μM DCFH-DA in serum-free medium was added and incubated for 20 min at 37 °C. Then the cells were washed 3 times with serum-free cell culture medium to remove DCFH-DA and analyzed by flow cytometry or microplate reader (λ_Excitation_ = 488 nm, λ_Emission_ = 525 nm). Data quantification was performed with FlowJo V10.

### 2.10. Mitochondrial Membrane Potential (MMP) Assay

Mitochondrial membrane potential JC-1 was detected using the mitochondrial membrane potential assay kit with JC-1 (Beyotime Biotechnology, Shanghai, China) according to the manufacturer’s instructions. The osteosarcoma cells were cultured with analogue 25 (2 μM and 4 μM) for 48 h. The treated cells were digested and collected by centrifugation at 1000× *g* for 5 min, and 6 × 10^5^ cells were resuspended and incubated with JC-1 working solution for 20 min at 37 °C. The cells were centrifuged at 600× *g* for 3 min, and after being washed twice with the staining buffer, they were centrifuged and subjected to flow cytometry analysis.

### 2.11. Intracellular Ferrous Iron (Fe^2+^) Determination

A total of 1 × 10^5^ cells/well were seeded into a 24-well plate for 24 h, then cultured with analogue 25 (2 μM and 4 μM) for 48 h. After washing with PBS twice, cells were incubated with 1 μM FerroOrange solution (MedChemExpress, Monmouth Junction, NJ, USA) for 1 h and analyzed using an inverted fluorescence microscope (ZEISS, Jena, Germany).

### 2.12. Lipid Peroxidation Assay

Lipid peroxidation was detected using the Lipid Peroxidation Assay Kit with BODIPY 581/591 C11 (Beyotime Biotechnology, Shanghai, China) according to the manufacturer’s instructions. A total of 1 × 10^5^ cells/well were seeded into a 24-well plate for 24 h, and then cultured with analogue 25 (2 μM and 4 μM) for 48 h. After washing with PBS twice, cells were incubated with 10 μM BODIPY581/591 C11 solution for 1 h and observed using an inverted fluorescence microscope (ZEISS, Jena, Germany).

### 2.13. Glutathione (GSH) Content Detection

GSH content was detected using the GSH and GSSG Assay Kit (Beyotime Biotechnology, Shanghai, China) according to the manufacturer’s instructions. The osteosarcoma cells were cultured with analogue 25 (2 μM and 4 μM) for 48 h, then collected and treated with protein removal reagent M solution, and vortexed and mixed thoroughly. Samples were then quickly frozen and thawed twice using liquid nitrogen and water at 37 °C, and then placed at 4 °C for 5 min. After that, the cells were centrifuged at 10,000× *g* for 10 min at 4 °C, and the supernatant was used for GSH content detection according to the instructions. The standard curve was plotted using the different absorbances measured by the standard substance. The total glutathione (GSSG concentration calculated by the standard curve × 2) and GSSG contents were calculated by the standard curve of the sample, and the GSH content was calculated using the following formula: GSH = Total Glutathione-GSSG × 2.

### 2.14. Animal Studies

Six-week-old BALB/C nude female mice were purchased from Beijing Vital River Laboratory (Beijing, China), in which 5 × 10^6^ U2OS cells were inoculated into the tibia bone marrow cavity to establish the osteosarcoma xenograft model. After one week, PBS, sanguinarine analogue 25 (3 mg/kg and 6 mg/kg), and sanguinarine (3 mg/kg and 6 mg/kg) were injected into the tail vein every 3 days, respectively. The body weights were recorded every 4 days during administration; after 24 days, the mice were sacrificed, the blood was collected for biochemical detection, and the heart, liver, spleen, lung and kidney of the mice were collected for histopathological examination. The tibial tumours were removed and the tumour weight and volume were measured, with the latter calculated using the following formula: Tumour volume (mm^3^) = a × b^2^/2 (a is the longest diameter of tumour; b is the shortest diameter of tumour). All animal experiments were performed in accordance with the Guiding Principles for the Care and Use of Laboratory Animals, and all experimental procedures were approved by the Institutional Experimental Animal Committee of Northwestern Polytechnical University (Xi’an, China).

### 2.15. H&E Staining and Immunohistochemical Analysis

The tumour tissues were fixed in 4% formaldehyde for 3 days, and then embedded in paraffin and sliced into 4 μm thick sections. H&E staining was used for histomorphological analysis. For immunohistochemistry analyses, sections were processed for antigen retrieval for 15 min and blocked in 3% BSA for 30 min. The sections were then incubated overnight with primary antibodies against Bcl-2 (1:100), GPX4 (1:200), and SLC7A11 (1:200) at 4 °C, and incubated with HRP-conjugated IgG (1:400) for 50 min after three washes with PBS. A DAB Horseradish Peroxidase Colour Development Kit was used to detect immunoactivity, followed by counterstaining with hematoxylin. Sections were examined under a fluorescence microscope (Nikon, Tokyo, Japan) and all sections scanned using an Aperio AT2 Digital Whole Slide Scanner (Leica, Wetzlar, Germany).

### 2.16. Serum Analysis

Serum was evaluated for alanine aminotransferase (ALT), aspartate transaminase (AST), urea nitrogen (BUN), and creatinine (CREA) content using assay kits, according to the manufacturer’s guidelines for each.

### 2.17. Statistical Analysis

All data are expressed as mean ± standard deviation. Statistical comparisons were made using Student’s *t* test or ANOVA; * *p* < 0.05 and ** *p* < 0.01 indicated statistical difference. GraphPad Prism 8 software was used for data analysis.

## 3. Results

### 3.1. Screening of Highly Active Sanguinarine Analogues to Inhibit the Growth of Osteosarcoma Cells

**Figure 1:**
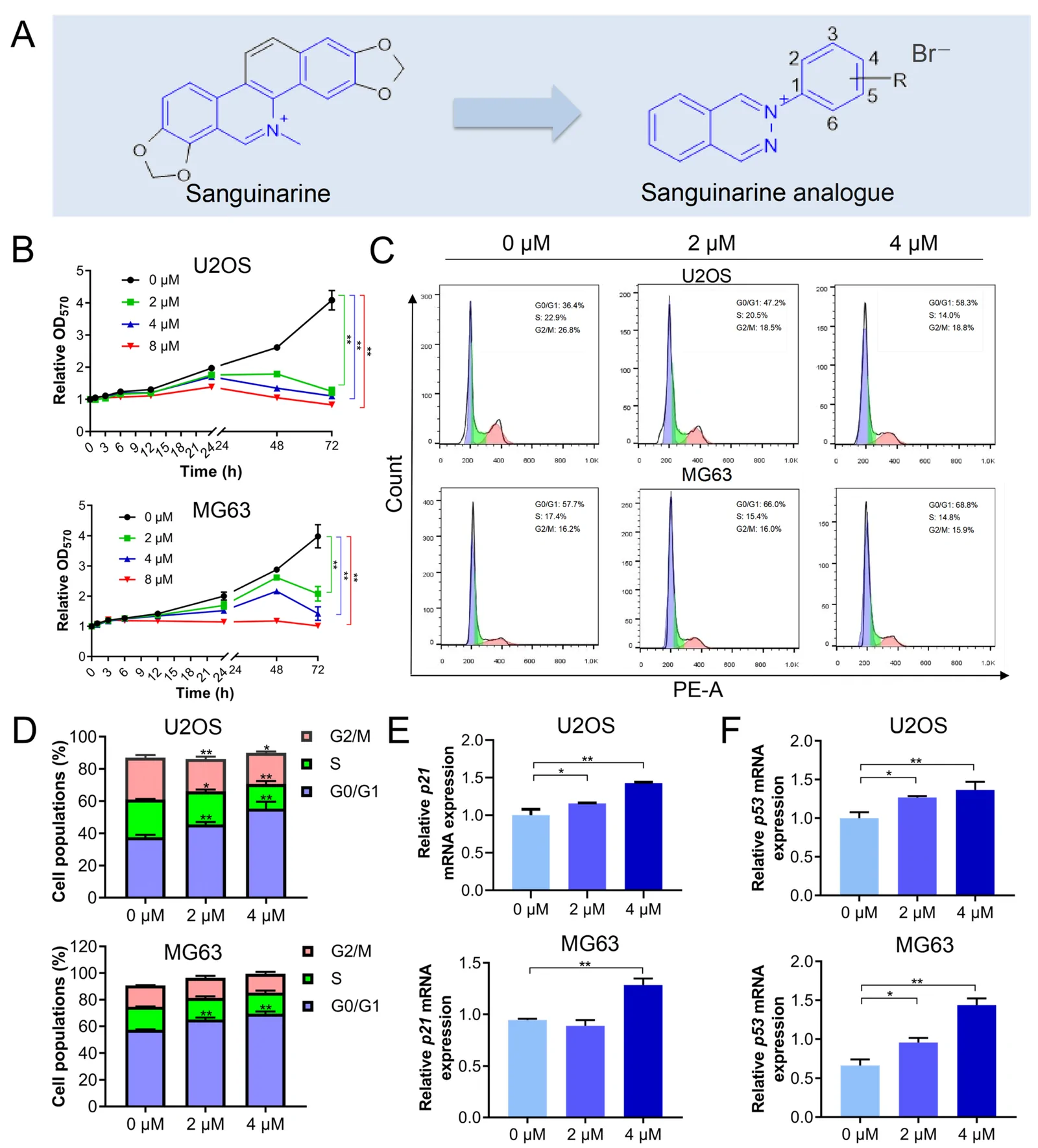
Analogue 25 inhibits the proliferation of osteosarcoma cells and arrests the cell cycle to G0/G1 stage. (**A**) The structural framework of the analogues of sanguinarine. (**B**) U2OS and MG63 cells were treated with different concentrations of analogue 25 (0, 2, 4, and 8 μM) for 1, 3, 6, 12, 24, 48, and 72 h, and cell viability was measured by MTT assay. (**C**) U2OS and MG63 cells were treated with analogue 25 for 48 h, cells were then stained with PI and measured by flow cytometry. Blue represents the G0/G1 phase, green represents the S phase, and pink represents the G2/M phase. (**D**) Quantitative analyses of cell cycle in (**C**). (**E**,**F**) Real-time PCR analysis of *p21* and *p53* expression in U2OS and MG63 cells treated with analogue 25 (0, 2, and 4 μM) (*n* = 3). The data are presented as the mean ± s.d.; * *p* < 0.05 and ** *p* < 0.01 versus control group.

Quaternary benzo[c]phenanthridine alkaloid (QBA) analogues, 2-phenylphthalazin-2-ium scaffold (Figure 1A) and its 30 kinds of derivatives were designed and synthesized previously by our team [15] (Table S1). First, we examined the inhibitory effect of analogues 1–30 on the proliferation of U2OS and MG63 cells by the MTT assay, with the aim of identifying effective and highly active compounds. The results showed that analogues 18, 19, 23, 25, 26, 27, 28, and 30 exhibited 50% inhibitory efficiency against U2OS cells when the concentration is below 20 μM (Figure S1), while analogues 19, 25, and 30 exhibited a 50% inhibition rate against MG63 cells (Figure S2). Therefore, we determined the median lethal concentrations for the analogue 19, 25, and 30 compounds. U2OS and MG63 cells were treated with 0, 0.0064, 0.032, 0.16, 0.8, 1.25, 2.5, 5, 10, 20, 100, 500, and 1000 μM of analogues 19, 25, and 30 for 48 h. The IC_50_ values of analogues 19, 25, and 30 in U2OS cells were 1.327, 1.484, and 1.800 μM, while in MG63 cells they were 1.941, 1.954, and 2.513 μM (Figure S3). Given the manifestations in the two types of osteosarcoma cells, analogue 25 (2-(2,3-Dichlorophenyl)phthalazin-2-ium Bromide) was used for the subsequent experiments, being the most effective inhibitor of cell proliferation.

U2OS and MG63 cells were treated with 0, 2, 4, and 8 μM of analogue 25 for 24, 48, and 72 h, respectively. After 24 h of treatment, the U2OS cells showed a slight proliferation, followed by cell death that was time- and concentration-dependent. Similar results were also observed in MG63 cells. The cell proliferation slowdown began at 48 h, and an 8 μM concentration treatment caused the cells to lose their proliferative ability (Figure 1B). Furthermore, we assessed whether analogue 25 could affect the cell cycle, with the results showing that analogue 25 could significantly increase the number of U2OS and MG63 cells in the G0/G1 phase and reduce the number of U2OS cells in the S and G2/M phases (Figure 1C,D). In addition, the expression levels of G1/S check point genes, *p21* and *p53*, were further analyzed. As shown in Figure 1E,F, as the concentration of analogue 25 increases, the expressions of *p21* and *p53* significantly increase, results consistent with those of the cell cycle detection. Therefore, analogue 25 exhibits a significant anti-proliferative effect on osteosarcoma cells and causes the cells to arrest at the G0/G1 phase.

### 3.2. Sanguinarine Analogue 25 Can Induce Apoptosis in Osteosarcoma Cells

**Figure 2:**
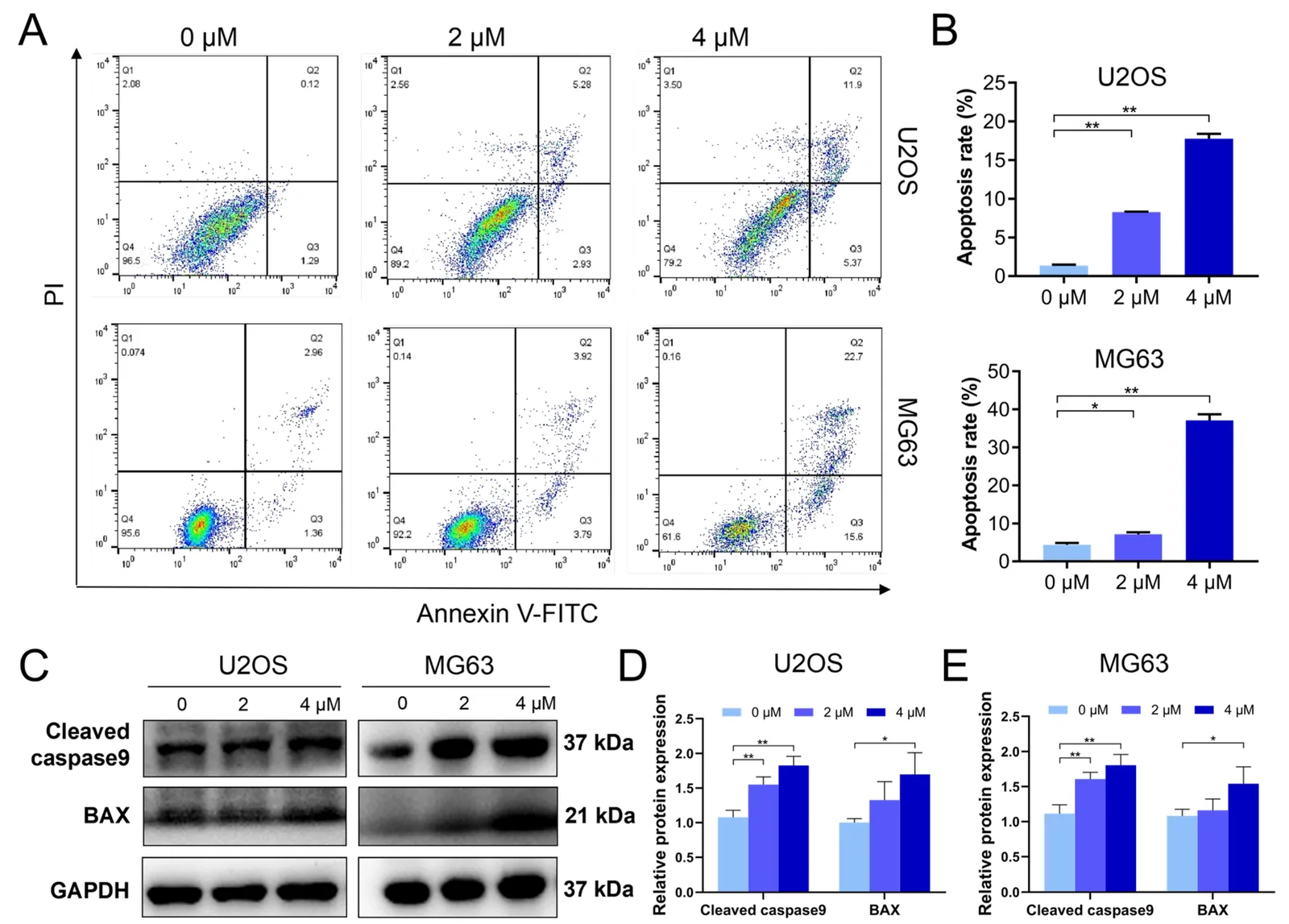
Analogue 25 promotes the apoptosis of osteosarcoma cells. (**A**,**B**) U2OS and MG63 cells were treated with analogue 25 (0, 2, and 4 μM) for 48 h and stained by PI and Annexin V-FITC, and analyzed by flow cytometry. (**C**) Western blotting analysis of Cleaved caspase9, BAX, and BCL-2 expression in U2OS and MG63 cells treated with analogue 25 (0, 2, and 4 μM). (**D**,**E**) Quantification of Cleaved caspase9, BAX, and BCL-2 levels versus GAPDH levels (*n* = 3). The data are presented as the mean ± s.d.; * *p* < 0.05 and ** *p* < 0.01 versus control group.

Given that analogue 25 could inhibit cell proliferation at high concentrations, we then investigated whether it could induce cell apoptosis. As shown in Figure 2A,B, analogue 25 significantly increased the apoptosis rate of U2OS and MG63 cells in a dose-dependent manner, indicating that it triggered apoptosis. Further analyzing the expression of apoptosis-related proteins revealed that analogue 25 dose-dependently increased cleaved caspase-9 and BAX levels (Figure 2C–E). The above results confirm that analogue 25 can induce apoptosis in osteosarcoma cells.

### 3.3. Sanguinarine Analogue 25 Modulates ROS Generation, MMP and Fe^2+^ Level in Osteosarcoma Cells

**Figure 3:**
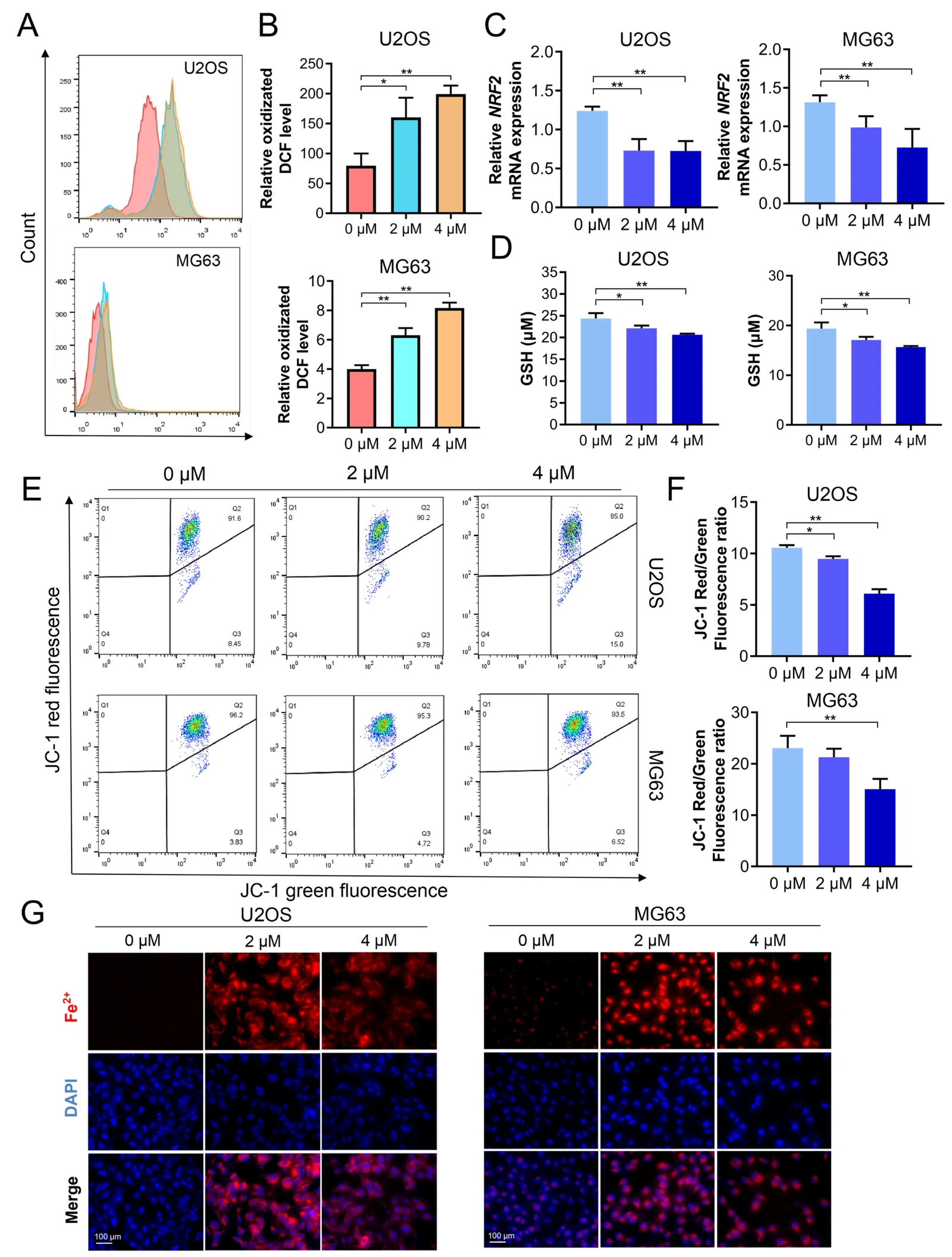
Analogue 25 modulates ROS generation, MMP and lipid peroxidation in osteosarcoma cells. (**A**,**B**) U2OS and MG63 cells treated with the analogue 25 (0, 2 and 4 μM) were detected for intracellular ROS levels by flow cytometry staining with DCF-DA. (**C**) Real-time PCR analysis of *Nrf2* expression in U2OS and MG63 cells treated with analogue 25 (0, 2, and 4 μM) (*n* = 3). (**D**) The content of GSH in U2OS and MG63 cells. (**E**,**F**) U2OS and MG63 cells were treated with analogue 25 for 48 h, cells were then stained by JC-1 and analyzed by flow cytometry. (**G**) Images of FerroOrange stained U2OS and MG63 cells treated with analogue 25 (0, 2, and 4 μM) (Scale bar = 100 μm). The data are presented as the mean ± s.d.; * *p* < 0.05 and ** *p* < 0.01 versus control group.

Excessive production of ROS can inhibit cancer cell proliferation [24], so we further investigated the effect of analogue 25 on the ROS level of osteosarcoma cells by DCFH-DA probe. Analogue 25 treatment could significantly induce intracellular ROS levels in U2OS and MG63 cells (Figure 3A,B); moreover, *Nrf2* expression and GSH concentration were downregulated in analogue 25-treated osteosarcoma cells (Figure 3C,D), and it could damage mitochondrial activity by reducing the MMP (Figure 3E,F). The FerroOrange (Fe^2+^ indicator) was used to detect the level of Fe^2+^ in osteosarcoma cells, and we found that analogue 25-treated cells showed an increase in Fe^2+^ level (Figure 3G). These findings indicate that sanguinarine analogue 25 induces ROS production and ferroptosis in osteosarcoma cells.

### 3.4. Sanguinarine Analogue 25 Can Regulate Mitochondrial-Mediated Apoptosis of Osteosarcoma Cells Through Regulating ROS Levels

**Figure 4:**
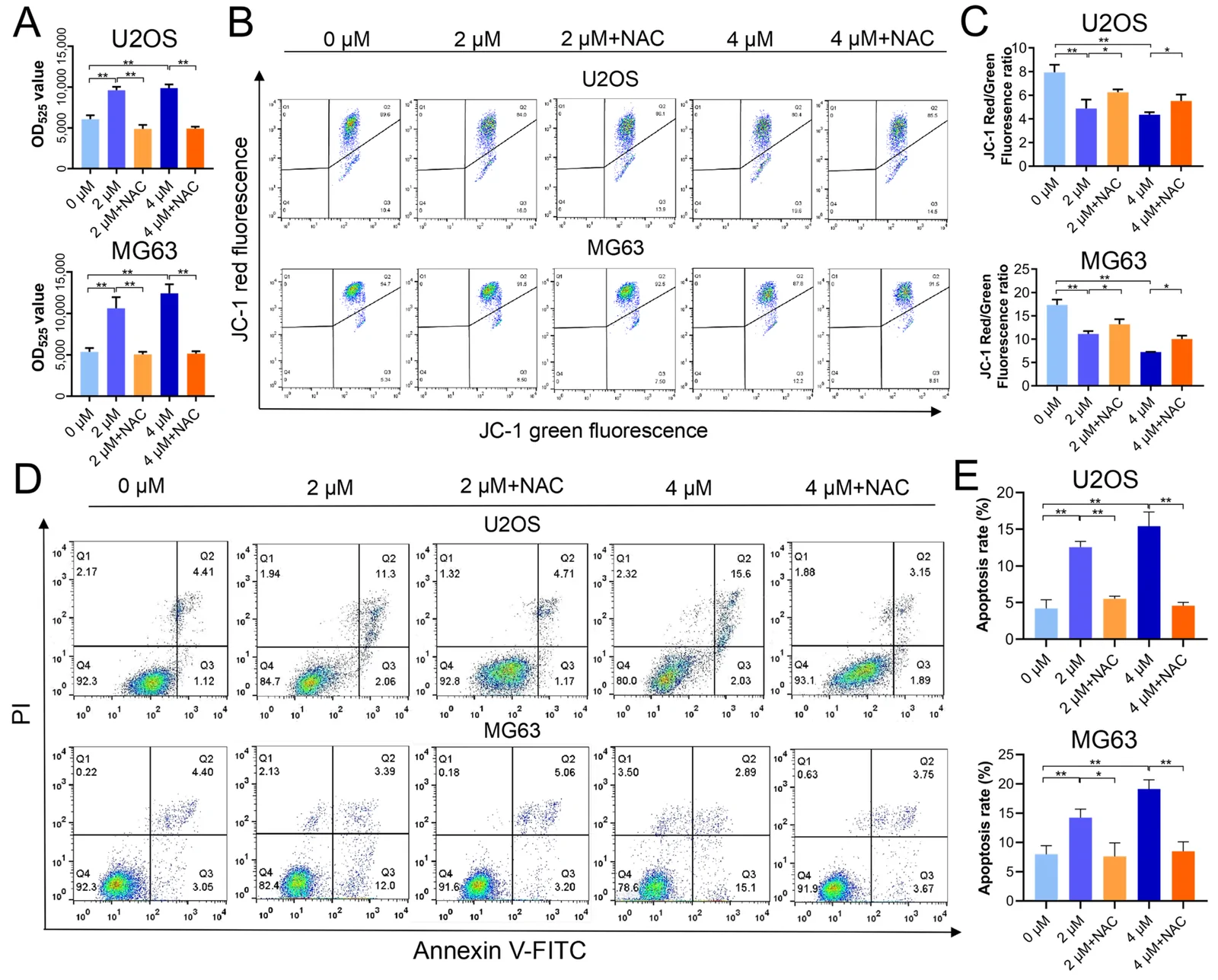
Analogue 25 regulates mitochondrial-mediated apoptosis of osteosarcoma cells through regulating ROS levels. (**A**) U2OS and MG63 cells treated with the analogue 25 (0, 2 and 4 μM) and 4 μM NAC, and absorbance value of cells were detected for intracellular ROS levels with DCF-DA staining. (**B**,**C**) U2OS and MG63 cells were treated with analogue 25 and 4 μM NAC for 48 h, cells were then stained by JC-1 and analyzed by flow cytometry. (**D**,**E**) U2OS and MG63 cells were treated with analogue 25 (0, 2, and 4 μM) and 4 μM NAC for 48 h and stained by PI and Annexin V-FITC, and analyzed by flow cytometry. The data are presented as the mean ± s.d.; * *p* < 0.05 and ** *p* < 0.01 versus control group.

Subsequently, we utilized N-Acetyl-L-cysteine (NAC) to study the effect of analogue 25 on osteosarcoma cells through ROS regulation. NAC is an intracellular ROS scavenger, which, when added to osteosarcoma cells, can significantly inhibit the increase in ROS levels caused by analogue 25 (Figure 4A). After detecting the MMP using the JC-1 fluorescent probe, it was found that NAC could reverse the changes in MMP in U2OS and MG63 cells, converting them from the green fluorescent monomer to the red fluorescent polymer form (Figure 4B,C). Furthermore, the results of the flow cytometry experiment revealed that NAC could reverse the promoting effect of analogue 25 on osteosarcoma cell apoptosis (Figure 4D,E). These results demonstrate that analogue 25 regulates the apoptosis of osteosarcoma cells by controlling intracellular ROS levels.

### 3.5. Sanguinarine Analogue 25 Can Affect Ferroptosis in Osteosarcoma Cells Through NRF2/GPX4 Pathway

**Figure 5:**
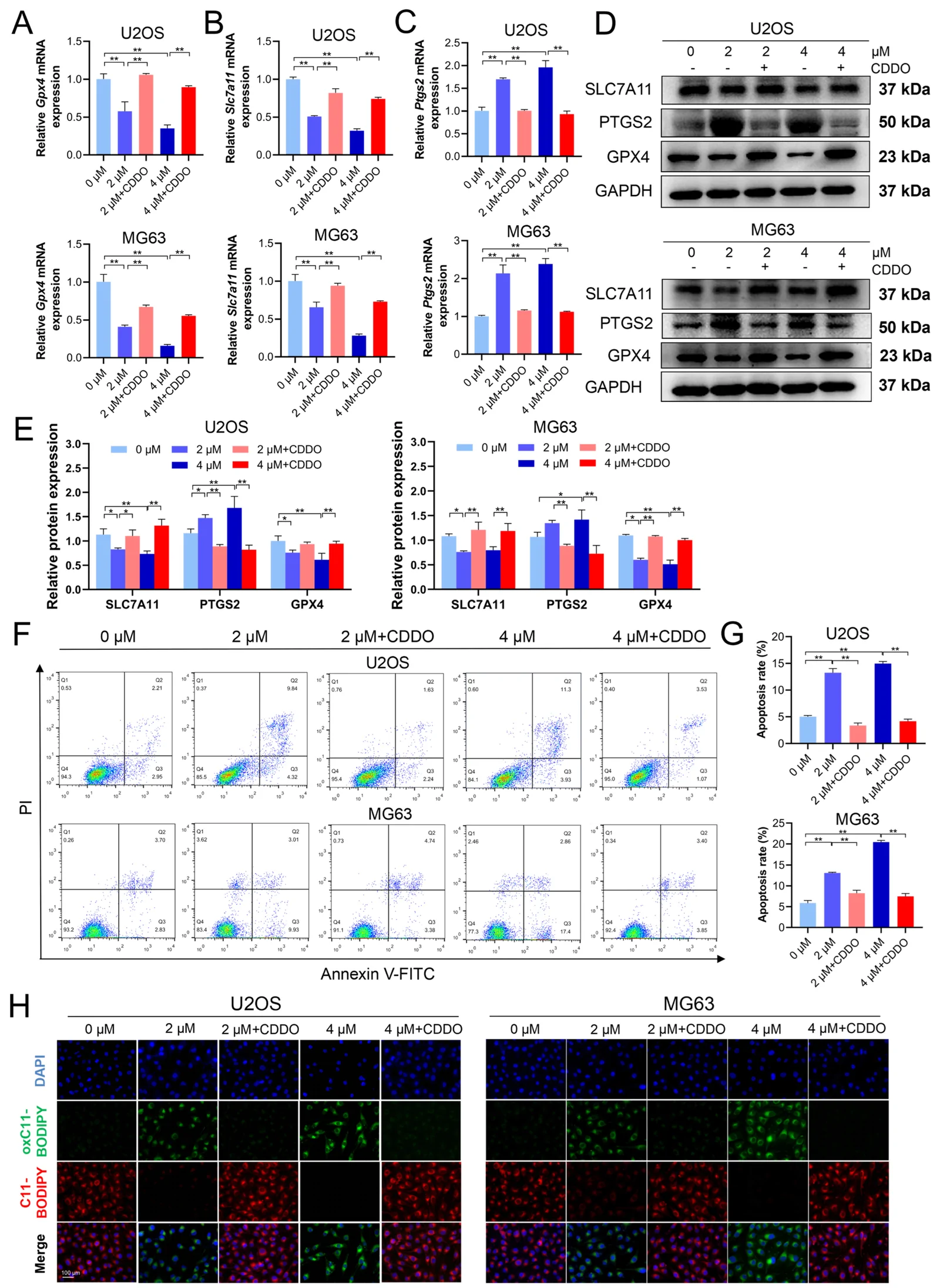
Analogue 25 affects ferroptosis in osteosarcoma cells through NRF2/GPX4 pathway. (**A**–**C**) Real-time PCR analysis of *Gpx4*, *Slc7a11*, and *Ptgs2* expression in U2OS and MG63 cells treated with analogue 25 (0, 2, and 4 μM) and 1 μM CDDO-Me (*n* = 3). (**D**,**E**) Western blotting analysis of GPX4, SLC7A11, and PTGS2 expression in U2OS and MG63 cells treated with analogue 25 (0, 2, and 4 μM) and 1 μM CDDO-Me (*n* = 3). (**F**,**G**) U2OS and MG63 cells were treated with analogue 25 (0, 2, and 4 μM) and 1 μM CDDO-Me for 48 h and stained by PI and Annexin V-FITC, and analyzed by flow cytometry. (**H**) Images of C11-BODIPY 581/591 stained U2OS and MG63 cells treated with analogue 25 (0, 2, and 4 μM) and 1 μM CDDO-Me for 48 h (Scale bar = 100 μm). Blue represents the DAPI, Green represents the oxC11−BODIPY, Red represents the C11−BODIPY. The data are presented as the mean ± s.d.; * *p* < 0.05 and ** *p* < 0.01 versus control group.

GPX4 can reduce intracellular lipid reactive oxygen species, is crucial for inhibiting ferroptosis [25], and is the transcriptional target of NRF2 [22]; therefore, we investigated the mechanism of action of analogue 25 on ferroptosis in osteosarcoma cells. Badoxolone methyl (CDDO-Me), a synthetic NRF2 activator, is utilized to regulate the NRF2/GPX4 pathway [23]. Firstly, we investigated the effects of different concentrations of analogue 25 and CDDO-Me on the expression of ferroptosis-related genes in U2OS and MG63 cells. The qPCR results showed that analogue 25 could inhibit the expression of *Gpx4* and *Slc7a11* and promote the expression of *Ptgs2* in a concentration-dependent manner. CDDO-Me could reverse the expression changes in ferroptosis-related genes (Figure 5A–C). The protein levels of GPX4, SLC7A11 and PTGS2 were affected by analogue 25 and CDDO-Me, consistent with the changes in mRNAs (Figure 5D,E). Furthermore, CDDO-Me also reversed the inhibitory effect of analogue 25 on apoptosis in U2OS and MG63 cells (Figure 5F,G), as well as its promoting effect on lipid peroxidation production (Figure 5H). These results indicate that analogue 25 can promote ferroptosis through the NRF2/GPX4 pathway.

### 3.6. Sanguinarine Analogue 25 Inhibits Osteosarcoma Growth by Inducing Apoptosis and Ferroptosis in Mice

**Figure 6:**
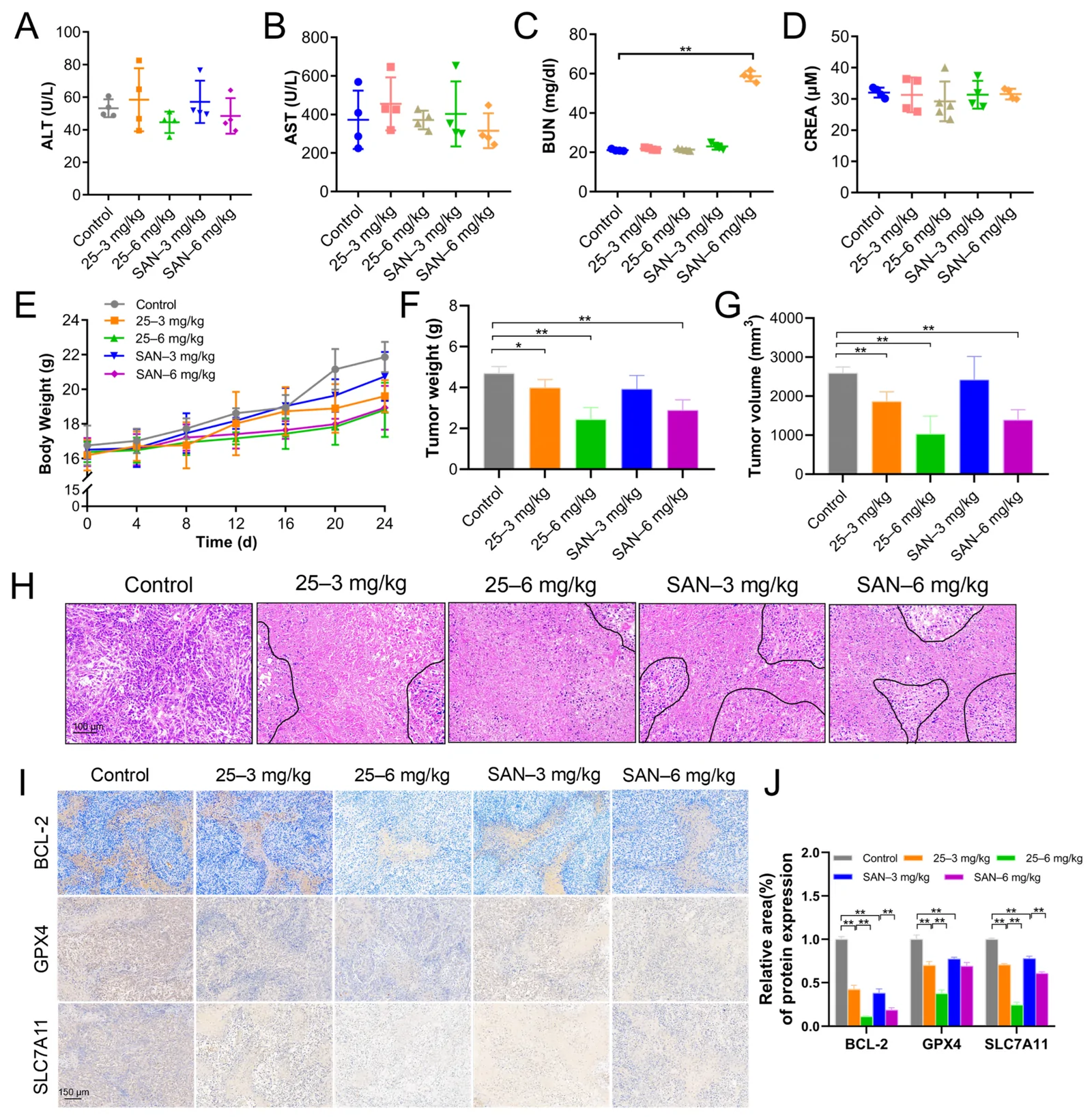
Analogue 25 inhibits osteosarcoma growth by inducing apoptosis and ferroptosis in mice. (**A**–**D**) ELISA analysis of ALT, ATS, BUN, and CREA levels in serum. (**E**) Female mice were treated with analogue 25 (3 and 6 mg/kg) and sanguinarine (3 and 6 mg/kg) for 24 days, body weight was measured (*n* = 5). (**F**,**G**) Tumor weight and tumor volume of mice were measured (*n* = 5), compared to the control group. (**H**) Representative H&E staining images of tumour tissue (Scale bar = 100 μm). (**I**,**J**) Area (%) of BCL-2, GPX4, and SLC7A11 expression in tumor tissues was determined by immunohistochemistry (Scale bar = 150 μm). The data are presented as the mean ± s.d.; * *p* < 0.05 and ** *p* < 0.01 versus control group.

To evaluate the therapeutic effect of sanguinarine analogue 25 on osteosarcoma, we established an in situ osteosarcoma model and intravenously administered sanguinarine analogue 25 (3 mg/kg and 6 mg/kg) and sanguinarine (3 mg/kg and 6 mg/kg) for 24 days. Serum analysis revealed that analogue 25 did not have any effect on the levels of ALT, AST, BUN, and CREA in the mice (Figure 6A–D); however, 6 mg/kg of sanguinarine increased the BUN content in the blood, indicating that it has certain renal toxicity. After the treatment, the tumour tissues of the mice were photographed and weighed. As shown in Figure 6E–G, the results showed that analogue 25 (6 mg/kg) could effectively inhibit tumour growth and was significantly more effective than the positive control group. Compared with the control group, the mice in the analogue 25 treatment group gained weight at a slower rate, indicating that it has a favourable therapeutic effect (Figure 6E). In addition, HE staining was performed on the osteosarcoma tissues of mice, with the results showing that analogue 25 caused extensive necrosis in these tissues (Figure 6H). Through immunohistochemical staining using antibodies targeting apoptosis- and ferroptosis-related proteins, the results indicated that analogue 25 inhibited BCL-2, GPX4 and SLC7A11 expression (Figure 6I,J). Taken together, these results demonstrate that analogue 25 can suppress osteosarcoma growth through regulating apoptosis and ferroptosis in vivo.

## 4. Discussion

In this study, we demonstrated that 2-(2,3-dichlorophenyl)phthalazin-2-ium bromide, sanguinarine analogue 25, exhibited anti-tumour effects on osteosarcoma cells U2OS and MG63. The underlying mechanism lies in the fact that analogue 25 activates the reactive oxygen pathway, resulting in increased ROS levels and lipid peroxidation, decreased GSH levels and MMP, and promotion of ferroptosis and apoptosis, thereby inhibiting the proliferation and normal cell cycle of osteosarcoma cells and suppressing osteosarcoma disease progression.

Sanguinarine, a benzophenanthridine alkaloid, is mainly extracted from the roots of *Sanguinaria canadensis*, and has unique advantages in terms of its antioxidation, anti-inflammation, antibacterial, and anticancer properties [26]. In ulcerative colitis, sanguinarine can reduce the levels of pro-inflammatory cytokines, which helps to increase colon length and body weight [27]. In recent years, research has indicated that sanguinarine disrupts mitochondrial function and induces apoptosis in osteosarcoma cells by upregulating single-stranded DNA-binding protein 1 (SSBP1), which inhibits the PI3K/Akt/mTOR signaling pathway [28]. In ER^+^ breast cancer, sanguinarine demonstrated anti-metastatic and anti-proliferative effects through the modulation of epithelial–mesenchymal transition (EMT)-related protein expression [29,30]. Moreover, sanguinarine has been shown to impede the migration and metastasis of colorectal cancer by inhibiting the Wnt/β-catenin signaling pathway and EMT process [31]. However, sanguinarine is highly soluble in organic solvents and has poor solubility in water, this limits its potential application in developing anticancer drugs [32]; therefore, we synthesized a series of sanguinarine analogues (1–30) based on the active groups of sanguinarine to address the issue of water solubility. Among them, analogues 26 and 27 exhibited excellent antifungal infection activity [15]. However, the mechanism of these analogues in osteosarcoma remains unclear.

Firstly, we conducted a screening of the effects of these 30 sanguinarine analogues on the proliferation of two types of osteosarcoma cells, U2OS and MG63. The results showed that analogue 25 had the greatest inhibitory effect on cell proliferation, with the IC_50_ values in U2OS and MG63 cells being 1.484 and 1.954 μM, respectively, and they exhibited a good concentration-dependent effect. Furthermore, we found that the intervention of sanguinarine analogue 25 caused osteosarcoma cells to be arrested in the G0/G1 phase, accompanied by the upregulation of cell cycle arrest regulatory genes *p53* and *p21*. We also observed that simulated analogue 25 significantly increased the osteosarcoma cell apoptosis rate, significantly upregulated the pro-apoptotic genes Caspase9 and BAX. In the in situ nude mouse osteosarcoma model, it was also demonstrated that analogue 25 could inhibit the growth of osteosarcoma.

Surprisingly, the flow cytometry results showed that analogue 25 increased the levels of ROS in U2OS and MG63 cells, while reducing the level of GSH and the expression of Nrf2. This imbalance between the generation of reactive oxygen and antioxidants indicates that analogue 25 promotes oxidative stress in osteosarcoma cells. Mitochondria are the main source of ROS; high levels of ROS can directly lead to a decrease in MMP, and play a crucial role in various tumours [33,34]. We also found that analogue 25 could reduce the MMP of osteosarcoma cells; therefore, we investigated whether eliminating ROS could affect the apoptotic effect of analogue 25 on osteosarcoma cells. We treated U2OS and MG63 cells with the antioxidant NAC, with the results showing that it could reverse the effects of analogue 25 on osteosarcoma cells, manifested as decreased levels of ROS, increased MMP, and reduced cell apoptosis.

Mitochondrial dysfunction and damage can trigger ferroptosis [35]. During this process, the interaction between ROS and ferrous ions leads to the outbreak of lipid peroxidation and triggers ferroptosis [36,37]. The intracellular level of Fe^2+^ and assessments of ferroptosis-related gene expression were conducted on U2OS and MG63 cells treated with analogue 25. We found that analogue 25 could increase Fe^2+^ levels and reduce the expressions of GPX4 and SLC7A11, indicating that it could trigger ferroptosis in osteosarcoma cells. GPX4 is an antioxidant enzyme that can catalyze the degradation of hydrogen peroxide and hydroperoxide and inhibit the generation of lipid ROS, and is a core molecule for inhibiting ferroptosis [25]. GSH serves as a crucial cofactor for this process, and its reduction results in diminished GPX4 activity [38]. In our study, we demonstrated that analogue 25 elicited a concentration-dependent reduction in both GSH levels and GPX4 expression in osteosarcoma cells. Furthermore, we discovered that analogue 25 could inhibit the expression of *Nrf2*, which is an important regulatory gene for GPX4 and SLC7A11 [22,39]; therefore, we proposed the hypothesis that analogue 25 may regulate ferroptosis in osteosarcoma cells through the Nrf2/GPX4 pathway. Upon activation of NRF2 via CDDO-Me [23], it was observed that the combination with CDDO-Me markedly attenuated the pro-apoptotic and lipid peroxidation effects induced by analogue 25 in U2OS and MG63 cells, and simultaneously restored the expression levels of GPX4 and SLC7A11. This indicates that the downregulation of NRF2, GPX4, and SLC7A11 plays a crucial role in ferroptosis induced by analogue 25 in osteosarcoma cells. Consistent with this, analogue 25 was found to suppress BCL-2, GPX4 and SLC7A11 expression in tumour tissue in the in situ osteosarcoma mouse model.

Overall, we report for the first time that the sanguinarine analogue 25, 2-(2,3-dichlorophenyl)phthalazin-2-ium bromide, exhibits ROS-dependent apoptosis and ferroptosis in osteosarcoma. Therefore, water-soluble analogue 25 can exert excellent anti-tumour effects. Increasing evidence indicates that sanguinarine analogues represent a promising intervention strategy in treating tumours [40]; however, the specific targets of these chemically synthesized analogues in tumour cell regulation still need to be further explored. Additionally, the precise pharmacological mechanisms also require further determination. Although in our study we demonstrated that analogue 25 has potential anti-tumour effects on osteosarcoma, namely achieved through ROS and Nrf2/GPX4 to induce apoptosis and ferroptosis, there are still some limitations. Firstly, although we have demonstrated that analogue 25 promotes cell apoptosis and ferroptosis at both the cellular and animal levels through ROS, including inhibiting GPX4, SLC7A11, and Nrf2, further exploration is needed to clarify the specific regulatory mechanisms of analogue 25 on these molecules, thereby determining more precise targets. Secondly, extensive research is necessary to compare the different targets and mechanisms of sanguinarine and its analogues in regulating osteosarcoma, so as to facilitate the further design and optimization of said sanguinarine analogues.

## 5. Conclusions

In summary, in this study, we evaluated the anti-tumour activity of 30 sanguinarine analogues. The results showed that analogue 25 exhibited significant activity against U2OS and MG63 cells, and was capable of inhibiting cell proliferation and arresting cells in the G0/G1 phase. Furthermore, analogue 25 could regulate cell apoptosis by increasing intracellular ROS and reducing MMP, and could also regulate ferroptosis-related protein expression (including GPX4, SLC7A11, and PTGS2) through the NRF2/GPX4 pathway, ultimately leading to lipid peroxidation accumulation and ferroptosis. Finally, in the U2OS orthotopic osteosarcoma model, analogue 25 demonstrated superior tumour growth inhibition effects without causing toxicity to mice. These results indicate that analogue 25 is a highly promising candidate drug that can be used to treat osteosarcoma by inducing apoptosis and ferroptosis.

## Figures and Tables

**Table 1:** Primer sequences used in qRT-PCR.

Gene	Primer Sequences	Accession No.
*p21*	Forward: 5′-CGATGGAACTTCGACTTTGTCA-3′	NM_000389.5
	Reverse: 5′-GCACAAGGGTACAAGACAGTG-3′	
*p53*	Forward: 5′-CAGCACATGACGGAGGTTGT-3′	NM_000546.6
	Reverse: 5′-TCATCCAAATACTCCACACGC-3′	
*Nrf2*	Forward: 5′-AGTTGTAGCCTCGGGAAGTTGTTAG-3′	NM_001145412.3
	Reverse: 5′-AGCCTCTTGGTTTCTGTCACTCATC-3′	
*Gpx4*	Forward: 5′-GAGGAGAGTAACTAC-3′	NM_001039847.3
	Reverse: 5′-CCGAACTGTTACAGGAA-3′	
*Slc7a11*	Forward: 5′-GGTCCATTACAGCTTTGTACG-3′	NM_014331.4
	Reverse: 5′-AATGTAGCTCCAATGCCAG-3′	
*Ptgs2*	Forward: 5′-TAAGTGCGATTGTACCCGGAC-3′	NM_000963.4
	Reverse: 5′-TTTGTAGCCATAGTCAGCATTGT-3′	
*Gapdh*	Forward: 5′-GGAGCGAGATCCCTCCAAAAT-3′	NM_001256799.3
	Reverse: 5′-GGCTGTTGTCATACTTCTCATGG-3′	

## Data Availability

The original contributions presented in this study are included in the article/Supplementary Materials. Further inquiries can be directed to the corresponding authors.

## Supplementary Materials

The following supporting information can be downloaded at: https://www.mdpi.com/article/10.3390/antiox15080941/s1, Table S1: The cationic structure of the sanguinarine analogue 1–30; Figure S1: The effects of analogues 1–30 on the proliferation of U2OS cells; Figure S2: The effects of analogues 1–30 on the proliferation of MG63 cells; Figure S3: The IC_50_ values of analogues 19, 25, and 30 in U2OS (A) and MG63 (B) cells.
