# Supplementary material for: A Sanguinarine Analogue Targeting ROS Signaling Exhibits Anti-Tumour Effects by Inducing Apoptosis and Ferroptosis in Osteosarcoma
Source: Antioxidants (Basel). 2026 Jul 29;15(8):941. doi: 10.3390/antiox15080941 (PMC13509277; doi:10.3390/antiox15080941)
Supplement: Supplementary file 1 [file antioxidants-15-00941-s001.zip › antioxidants-4414464-supplementary.pdf]

Supporting Information

A sanguinarine analogue targeting ROS signaling exhibits anti-tumour effects by inducing apoptosis and ferroptosis in osteosarcoma

Hui Zhang, Fangjun Cao, Jiaxin Wang, Chenxi Yun, Airong Qian*, Xiao Lin*

Experimental Methods

Cell viability assay

U2OS and MG63 cells (3×104 cells/well) were cultured in 96-well plates with 100 μL complete medium for 24 h; then, sanguinarine analogues 1-30 at different concentrations (0, 1, 5, 10, 15, and 20 μM) were added for 48 h. For the detection of IC_{50}, U2OS and MG63 cells were treated with 0, 0.0064, 0.032, 0.16, 0.8, 1.25, 2.5, 5, 10, 20, 100, 500, and 1000 μM of analogues 19, 25, and 30 for 48 h. A total of 20 μL MTT solution (5 mg/mL) was added to each well; then, the wells were incubated for 4 h at 37°C in the dark. The supernatant was removed and 120 μL DMSO was added to each well to fully dissolve the crystals. After incubation at 37℃ for 10 min with shaking, the absorbance value at 570 nm was detected by a Bio-Red microplate reader (Hercules, CA).

Statistical analyses

All numerical values are presented as means ± standard deviations. The statistical analyses were mainly conducted by Student's *t*-test, and statistical significance was accepted at $*p < 0.05$ and $**p < 0.01$. All statistical analyses were performed using GraphPad Prism 8.

Table S1. The cationic structure of the sanguinarine analogue 1-30.

Compound			Compound		
No.	R	Cationic structure	No.	R	Cationic structure
1	H	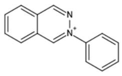	16	3'-OMe	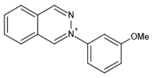
2	2'-F	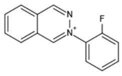	17	4'-OMe	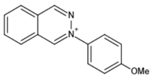
3	3'-F	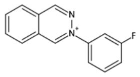	18	2'-CF_3	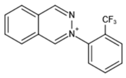
4	4'-F	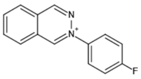	19	3'-CF_3	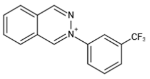
5	2'-Cl	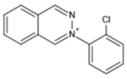	20	4'-CF_3	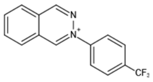
6	3'-Cl	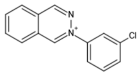	21	3'-CN	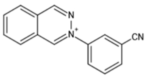
7	4'-Cl	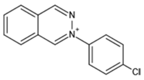	22	2'-NO_2	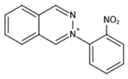
8	2'-Br	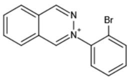	23	3'-NO_2	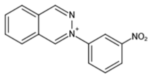
9	3'-Br	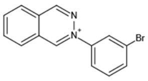	24	4'-NO_2	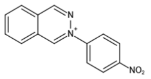
10	4'-Br	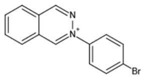	25	2',3'-diCl	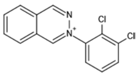
11	4'-I	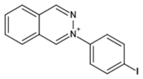	26	2',4'-diCl	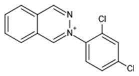
12	2'-CH_3	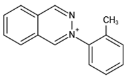	27	2',5'-diCl	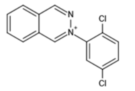
13	3'-CH_3	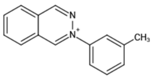	28	2',6'-diCl	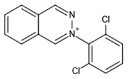
14	4'-CH_3	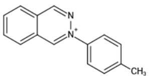	29	3',4'-diCl	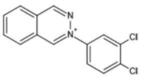
15	4'-Et	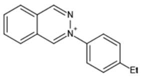	30	3',5'-diCl	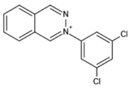

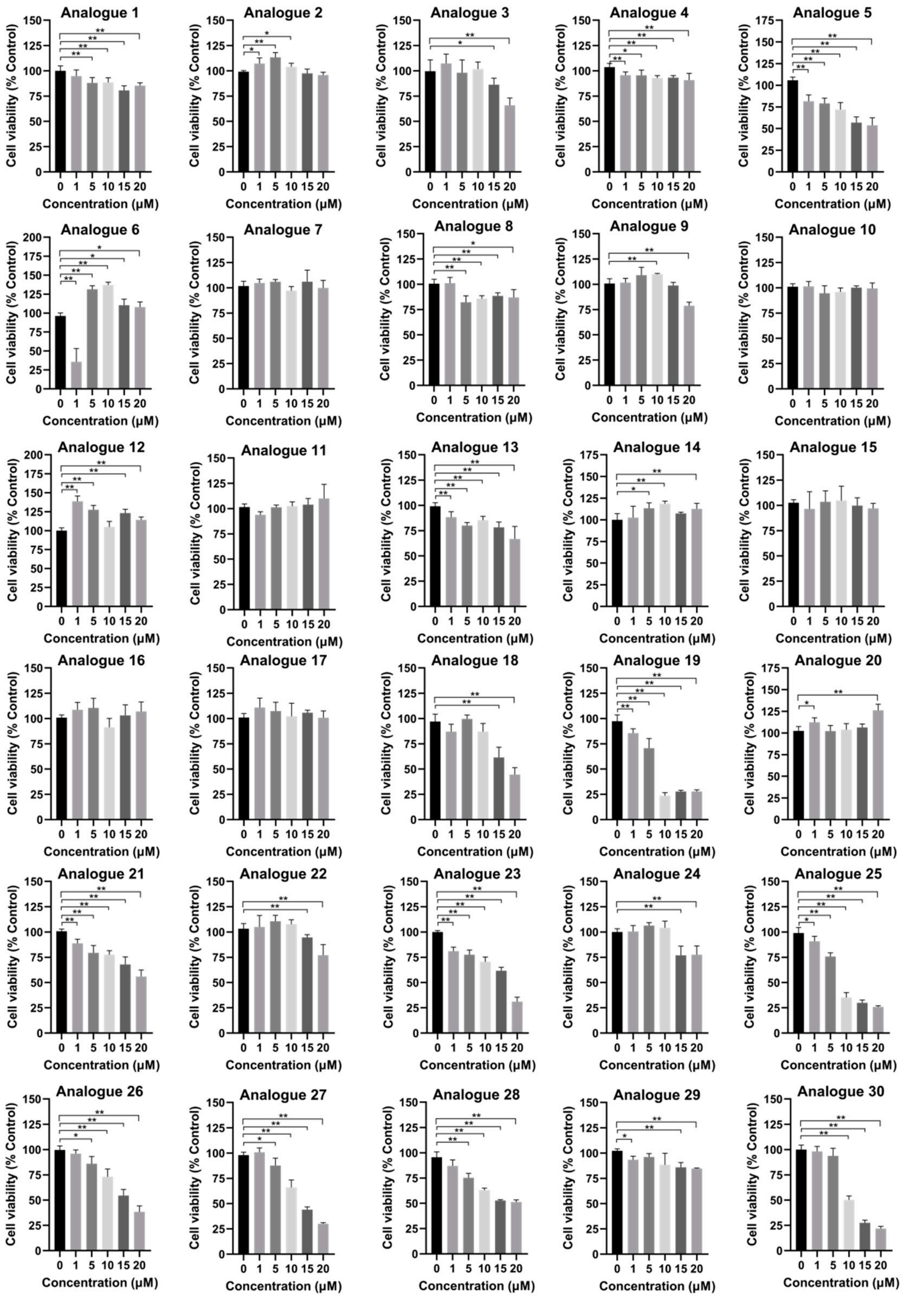

Figure S1. The effects of analogues 1-30 on the proliferation of U2OS cells.

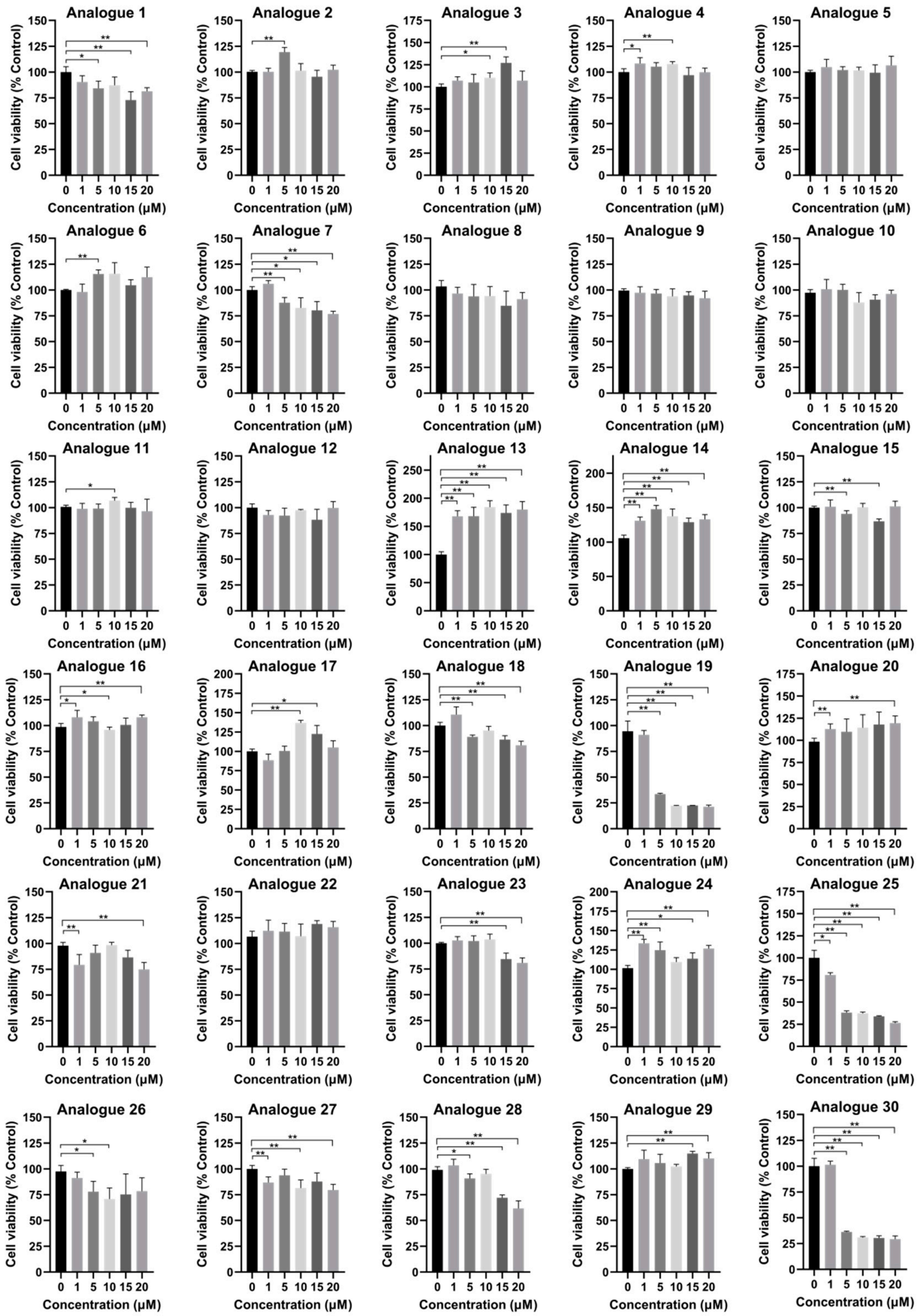

Figure S2. The effects of analogues 1-30 on the proliferation of MG63 cells.

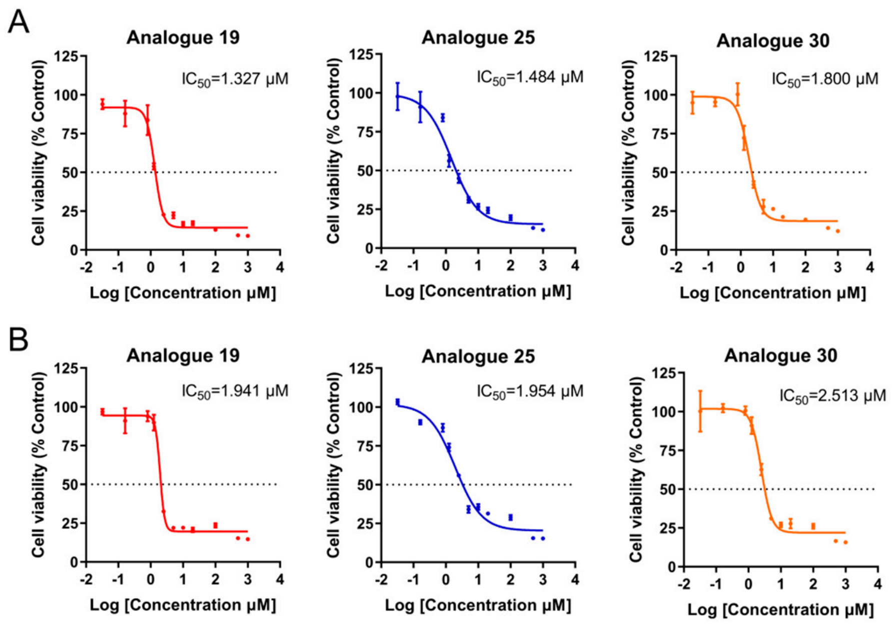

Figure S3. The IC_{50} values of analogues 19, 25, and 30 in U2OS (A) and MG63 (B) cells.
